# Association between Active Cancer and Risk of Thrombotic and Cardiovascular Outcomes in Outpatients with COVID-19: A CORONA-VTE Network Analysis

**DOI:** 10.1055/a-2713-2715

**Published:** 2025-10-17

**Authors:** Bridget McGonagle, Christie Greason, Darsiya Krishnathasan, Giovanni Scimeca, Antoine Bejjani, Candrika D. Khairani, Nada Hamade, Alyssa Sato, Hannah Leyva, Umberto Campia, Julia Davies, Nicole Porio, Ali A. Assi, Andre Armero, Anthony Tristani, Marcos D. Ortiz-Rios, Victor Nauffal, Zaid Almarzooq, Eric Wei, Valeria Zuluaga-Sánchez, Mehrdad Zarghami, Aditya Achanta, Sirus J. Jesudasen, Bruce C. Tiu, Geno J. Merli, Orly Leiva, John Fanikos, Aditya Sharma, Samantha Rizzo, Mariana B. Pfeferman, Ruth B. Morrison, Alec Vishnevsky, Judith Hsia, Mark R. Nehler, James Welker, Marc P. Bonaca, Brett J. Carroll, Samuel Z. Goldhaber, Zhou Lan, Behnood Bikdeli, Gregory Piazza

**Affiliations:** 1 Division of Cardiovascular Medicine, Brigham and Women's Hospital, Harvard Medical School, Boston, Massachusetts, United States; 2 Thrombosis Research Group, Brigham and Women's Hospital, Harvard Medical School, Boston, Massachusetts, United States; 3 Department of Medicine, Mount Auburn Hospital/Beth Israel Lahey Health, Cambridge, Massachusetts, United States; 4 Department of Medicine, Brigham and Women's Hospital, Harvard Medical School, Boston, Massachusetts, United States; 5 Department of Medicine, Massachusetts General Hospital, Boston, Massachusetts, United States; 6 Division of Pulmonary and Critical Care Medicine, Massachusetts General Hospital, Boston, Massachusetts, United States; 7 Department of Cardiology, Thomas Jefferson University Hospital, Philadelphia, Pennsylvania, United States; 8 Department of Pharmacy, Brigham and Women’s Hospital, Harvard Medical School, Boston, Massachusetts, United States; 9 Department of Medicine, Cardiovascular Medicine, University of Virginia Health, Charlottesville, Virginia, United States; 10 CPC Clinical Research, Aurora, Colorado, United States; 11 Department of Medicine, University of Colorado, Aurora, Colorado, United States; 12 Department of Medicine, Anne Arundel Research Institute, Annapolis, Maryland, United States; 13 Smith Center for Cardiovascular Outcomes Research, Division of Cardiology, Beth Israel Deaconess Medical Center, Harvard Medical School, Boston, Massachusetts, United States; 14 Center for Clinical Investigation, Brigham and Women's Hospital, Harvard Medical School, Boston, Massachusetts, United States; 15 Yale New Haven Hospital/Yale Center for Outcomes Research and Evaluation (CORE), New Haven, Connecticut, United States

**Keywords:** cancer, cardiovascular events, COVID-19, deep vein thrombosis, pulmonary embolism

## Abstract

Coronavirus disease 2019 (COVID-19) and active cancer are each independently associated with excess risk of thrombotic and cardiovascular events. However, data are limited regarding the risk of these events in outpatients with active cancer and concomitant COVID-19.

**Objectives:**

This study aimed to retrospectively examine the association between active cancer and thrombotic and cardiovascular outcomes among non-hospitalized patients with COVID-19.

**Methods:**

Data from the outpatient cohort with confirmed COVID-19 from the 10,420-patient multicenter U.S. CORONA-VTE Network registry were used. Active cancer was defined as having a malignancy diagnosis (excluding non-melanoma skin cancer) or receiving cancer-related treatment within the past year. Outcomes were independently adjudicated and included a composite of venous and arterial thromboembolism, and a composite of major adverse cardiovascular events, including thromboembolism, heart failure, myocarditis, new atrial fibrillation, and cardiovascular death within 90 days of COVID-19 diagnosis.

**Results:**

The registry included 6,576 outpatients, of whom 166 (2.5%) had active cancer (mean age 61 ± 16, 53% female). For outpatients with and without active cancer, the 90-day cumulative incidences of thromboembolism after developing COVID-19 were 4.2% and 1.2%, respectively (hazard ratio [HR]: 3.65; 95% confidence interval [CI]: 1.73–7.69, *p* < 0.001). Corresponding 90-day cumulative incidences of cardiovascular events were 5.4% and 1.9% (HR: 2.97; 95% CI: 1.46–6.05, *p* = 0.003). In adjusted analyses, non-hospitalized patients displayed an increased risk of thrombotic outcomes (HR: 2.48, 95% CI: 1.13–5.45, *p* = 0.024) but not cardiovascular outcomes (HR: 1.76, 95% CI: 0.85–3.62, *p* = 0.13).

**Conclusion:**

Outpatients with COVID-19 and active cancer demonstrated an increased hazard of thrombotic events compared with outpatients without cancer.

## Introduction

Cancer-associated thrombosis, which affects approximately 15% of patients with cancer, corresponds with poor overall prognosis and may complicate cancer-related treatment. 1 The pathophysiology underlying cancer-related thrombosis is complex and includes increased tissue factor expression by tumor and endothelial cells, production of tumor-secreted heparinases and tissue factor-laden microparticles, increased platelet count and activity, as well as reduced hepatic clearance of coagulation factors. 1 2 3 4 5 In patients with active cancer, the risk of adverse cardiovascular outcomes beyond thrombosis, such as heart failure and atrial fibrillation, is increasingly recognized as clinically important. 6 7 8 9

Coronavirus disease 2019 (COVID-19) is associated with an increased risk of various adverse cardiovascular events, including heart failure, myopericarditis, and arrhythmias, along with thromboembolic disease. 8 COVID-19 contributes to hypercoagulability via platelet hyperactivity, heightened levels of coagulation factors, acquired antiphospholipid antibodies, and reduced levels of endogenous anticoagulant proteins. 10 11

Concomitant COVID-19 in patients with active cancer has been associated with adverse outcomes, including increased short-term mortality. 12 In hospitalized patients with active cancer, concomitant COVID-19 has been associated with a nearly 15% risk of thrombotic complications by 30 days. 13 Among non-hospitalized patients with cancer, the impact of COVID-19 on cardiovascular events, including arterial and venous thrombotic events, remains unclear. 14 15 16 Given that the majority of patients with cancer are treated outside of the hospitalized setting, 3 we set out to define the risk of thrombotic and cardiovascular outcomes associated with active cancer and COVID-19 in outpatients.

## Methods

### Study Design

The multicenter CORONA-VTE Network registry included 10,420 patients with a confirmed diagnosis of severe acute respiratory syndrome coronavirus 2 (SARS-CoV-2) by reverse transcriptase polymerase chain reaction (RT-PCR) test. Patients were included from six different hospital systems across the United States, including the Mass General Brigham (MGB) Health system, Beth Israel Deaconess Medical Center, Anna Arundel Medical Center, University of Virginia Medical Center, University of Colorado Health System, and Thomas Jefferson University Hospital, between March 2020 and June 2022. 17 The study was approved by the Institutional Review Board at all participating sites, and the requirement to obtain informed consent was waived. Patient data were retrospectively collected via manual chart review of electronic health records. The design of the CORONA-VTE Network registry has been described in prior publications. 15 17

### Study Population

Adult patients (18 years and older) with RT-PCR confirmed COVID-19 were eligible for inclusion. Data from the outpatient cohort ( *n* = 6,576) were used for this analysis. Outpatient status was defined as participants not admitted to the hospital within 1 day of a positive COVID-19 test. Inpatients, including those admitted to the hospital within 1 day of a positive COVID-19 test, were excluded from our analysis because they are likely at an increased risk of experiencing thrombotic and cardiovascular events due to additional risk factors related to their hospitalization. Adjustment for age, sex, smoking status, history of cardiovascular disease, history of hemodialysis, and prior venous thromboembolism (VTE) may not sufficiently account for the disproportionate risk of thrombotic and cardiovascular events to which inpatients are exposed. A history of cardiovascular disease is defined as a history of heart failure, coronary artery disease, peripheral artery disease, atrial fibrillation, valvular heart disease, and a history of cerebrovascular events. Active cancer was defined as having either a recent diagnosis of malignancy (excluding non-melanoma skin cancer) within the past year and/or receiving cancer-related treatment within 1 year of COVID-19 diagnosis. Additional details related to cancer types and treatment were ascertained for outpatients who received care from the MGB health system. Cancer types were broadly categorized into eight different groups: Hematological, breast, urinary tract, lung, gastrointestinal tract, skin, gynecological, and others. Cancer treatment types were extracted up to 1 year prior to COVID-19 infection and were categorized as chemotherapy, immunotherapy, radiation, targeted/hormonal therapy, and surgical resection.

### Outcomes

Outcomes were collected up to 90 days post-COVID-19 diagnosis. The two main outcomes of interest were a composite of arterial and venous thrombotic events and a composite of major adverse cardiovascular events. Thrombotic outcomes included a composite of VTE (deep vein thrombosis [DVT] and pulmonary embolism [PE]), superficial vein thrombosis, and catheter-related thrombosis, as well as arterial thrombotic events, which included type I myocardial infarction, ischemic stroke or transient ischemic attack, major acute limb ischemia, and thrombotic-related death. Cardiovascular events included thrombotic events, heart failure, myocarditis, newly diagnosed atrial fibrillation, and cardiovascular-related death. Outcomes were prespecified and adjudicated by independent physicians using standardized definitions. 17

### Statistical Analysis

Demographics and baseline characteristics were reported among outpatients with and without cancer. Categorical variables were reported as frequency counts and percentages, while continuous variables were reported as means and standard deviations.

Non-thrombotic-related deaths and non-cardiovascular-related deaths were considered competing risks for composite thrombotic and cardiovascular events, respectively. Ninety-day cumulative incidence for thrombotic and cardiovascular outcomes and the respective 95% confidence intervals (CIs) were reported using time-to-first-event analysis based on Fine–Gray regression models. 18 For patients with versus without cancer, we reported unadjusted hazard ratios (HRs) and adjusted HRs based on a multivariable model adjusted for age, sex, smoking status, history of cardiovascular disease, history of hemodialysis, and prior VTE. The robust standard error accounted for the clustering of observations within sites. 19 A sensitivity analysis was conducted by excluding superficial vein thrombosis from the composite thrombotic and cardiovascular outcomes. Statistical analysis was conducted using R software (R for Linux; version 4.2.0; R Core Team 2022). Results were deemed statistically significant at *p* < 0.05.

## Results

### Patient Characteristics

**Fig. 1:**
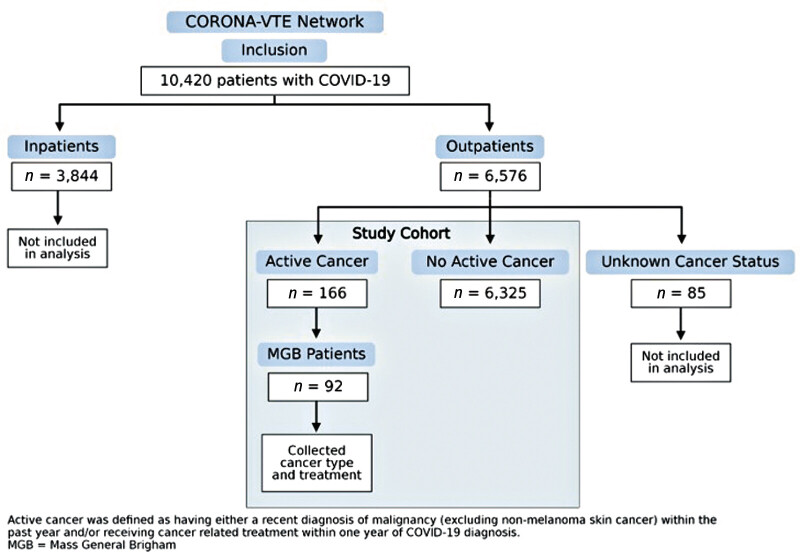
Study cohort.

The CORONA-VTE Network Registry included 10,420 participants, of whom 6,576 were outpatients. Within the outpatient cohort, 166 (2.5%) patients had active cancer, and 6,325 (96.2%) patients did not. Among outpatients, 85 (1.3%) had an unknown cancer status and were not included in the final analyses ( Fig. 1 ). The cohort of outpatients without active cancer had a mean age of 48 ± 18 years, while the cohort of those with active cancer had a mean age of 61 ± 16 years. Additional baseline characteristics for all patients are detailed in Table 1 .

**Table 1:** Baseline characteristics and COVID-19-related therapies

Variable	Outpatients without active cancer ( *n* = 6,325)	Outpatients with active cancer ( *n* = 166)
Age (years, SD)	47.6 ± 17.8	61.3 ± 16.0
Female sex (%)	60.3	53.0
Hispanic/Latinx (%)	22.0	13.9
Race (%)		
White	56.4	70.5
Black	17.8	12.0
Asian	3.1	4.2
Other	16.4	9.7
Not reported	6.3	3.6
Body mass index (kg/m ^2^ , SD)	30.2 ± 7.1	29.4 ± 6.6
Prior VTE (%)	3.3	11.4
Family history of VTE (%)	1.0	1.8
Current smoker (%)	5.3	3.0
Diabetes (%)	15.6	21.7
Hypertension (%)	32.3	54.8
History of coronary disease (%)	4.9	9.6
History of peripheral artery disease (%)	1.4	1.8
History of stroke or TIA (%)	3.1	7.2
Heart of heart failure (%)	1.1	2.4
Hemodialysis (%)	0.5	1.2
Baseline dual antiplatelet therapy a b (%)	0.8	0.0
Baseline use of anticoagulation b (%)	3.8	16.4
Therapies (new use after COVID-19; %)		
Corticosteroids	4.3	15.7
Antiviral agents	4.3	12.0
Anticoagulants	10.8	28.9
Antiplatelet agents	8.6	16.2
Vaccination status against SARS-CoV-2 c (%)		
Unvaccinated	89.2	76.5
Single dose	0.9	3.0
Two doses	4.6	6.6
More than two doses	5.2	13.9

Abbreviations: COVID-19, coronavirus disease 2019; SARS-CoV-2, severe acute respiratory syndrome coronavirus 2; SD, standard deviation; TIA, transient ischemic attack; VTE, venous thromboembolism.

a Cleaned data available only for patients from the Mass General Brigham health system ( *N* = 5,518).

b Baseline indicates use prior to COVID-19 diagnosis.

c Only includes patients with COVID-19 after vaccines were made available (COVID-19 diagnosis after December 14, 2020).

### Cancer-Specific Characteristics

**Fig. 2:**
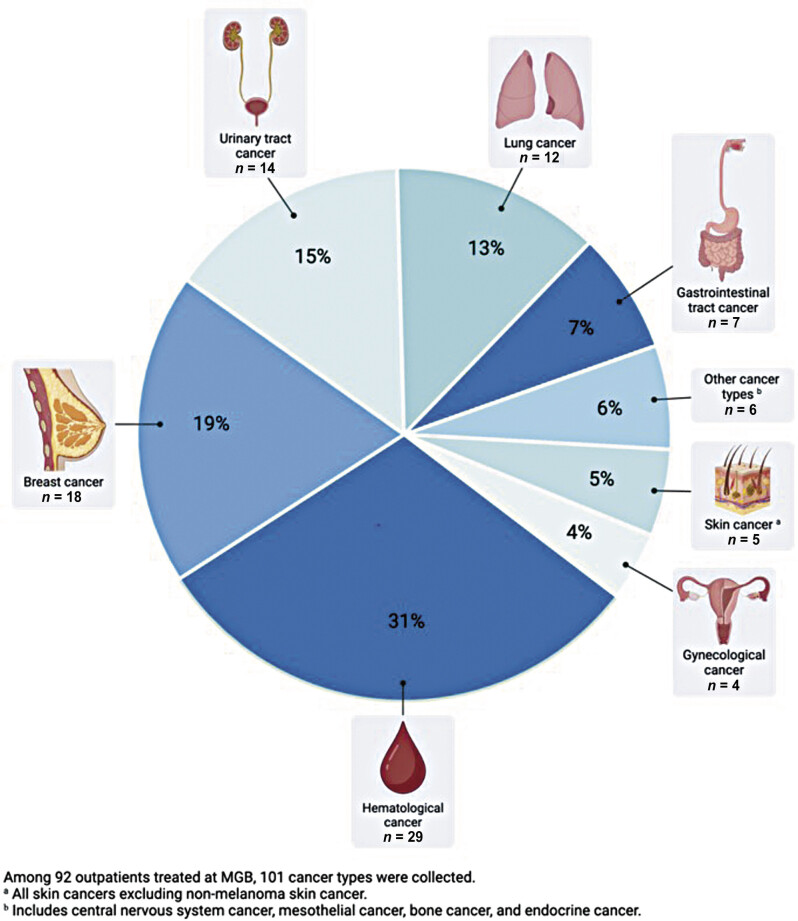
Frequencies of cancer types of outpatients treated at Mass General Brigham.

**Fig. 3:**
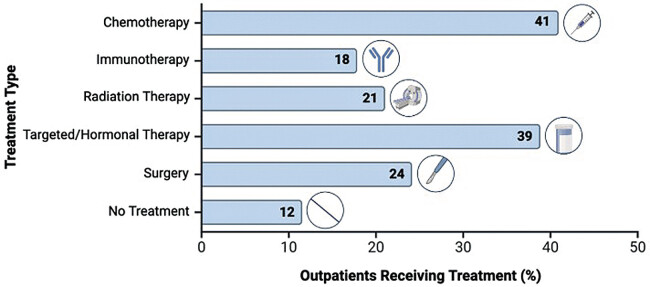
Frequencies of cancer treatment modalities received by outpatients with active cancer from Mass General Brigham.

Data regarding cancer types and treatments were restricted to the 92 patients who received care through the MGB health system. The most prevalent cancers within this population were hematological cancer (31%), breast cancer (19%), and urinary tract cancer (15%; Fig. 2 ). Cancer treatment types were collected up to 1 year prior to COVID-19 infection and were categorized as chemotherapy, immunotherapy, radiation, targeted/hormonal therapy, and/or surgical resection ( Fig. 3 ). The most common cancer treatments received were chemotherapy (41%) and targeted/hormonal therapies (39%).

### Main Outcomes

During the 90-day follow-up period, the cumulative incidences of thrombotic outcomes among outpatients with and without active cancer were 4.2% (95% CI: 2.05–8.74) and 1.2% (95% CI: 0.94–1.48), respectively (HR: 3.65; 95% CI: 1.73–7.69, *p* < 0.001; Table 2 ). After adjusting for covariates, outpatients with active cancer and COVID-19 had an increased hazard of thrombotic outcomes by 90-day follow-up (adjusted HR: 2.48, 95% CI: 1.13–5.45, *p* = 0.024).

**Table 2:** Summary of hazard ratios for outpatients with and without active cancer

Outcome	HR (95% CI)	*p* -Value
Thrombotic outcome composite		
Unadjusted	3.65	<0.001
Adjusted a	2.48	0.024
Cardiovascular composite		
Unadjusted	2.97	0.003
Adjusted a	1.76	0.13

Abbreviations: CI, confidence interval; HR, hazard ratio.

a Adjusted for age, sex, smoking, history of cardiovascular disease, history of hemodialysis, and history of prior venous thromboembolism.

The cumulative incidences of cardiovascular events for outpatients with versus without active cancer were 5.4% (95% CI: 2.88–10.26) and 1.9% (95% CI: 1.56–2.24), respectively (HR: 2.97; 95% CI: 1.46–6.05, *p* = 0.003). After adjusting for covariates, the hazard of cardiovascular events at 90 days was not significantly greater in outpatients with active cancer and COVID-19 compared with those without active cancer (adjusted HR: 1.76, 95% CI: 0.85–3.62, *p* = 0.13). At 90-day follow-up, DVT and PE were the most common individual outcomes for both patients with active cancer (DVT 28.6% and PE 28.6%) and without active cancer (DVT 26.0% and PE 30.1%; Supplementary Table S1 ).

### Sensitivity Analysis

Sensitivity analysis after exclusion of superficial vein thrombosis did not substantively change the main findings. Adjusted 90-day cumulative incidence rates of thrombotic events among outpatients with versus without active cancer were 3.6% (95% CI: 1.65–7.95%) and 1.1% (95% CI: 0.85–1.37%), respectively. The cumulative incidence rates of cardiovascular events were 5.4% (95% CI: 2.88–10.26%) and 1.9% (95% CI: 1.56–2.24) among outpatients with and without active cancer, respectively ( Supplementary Table S2 ).

## Discussion

**Fig. 4:**
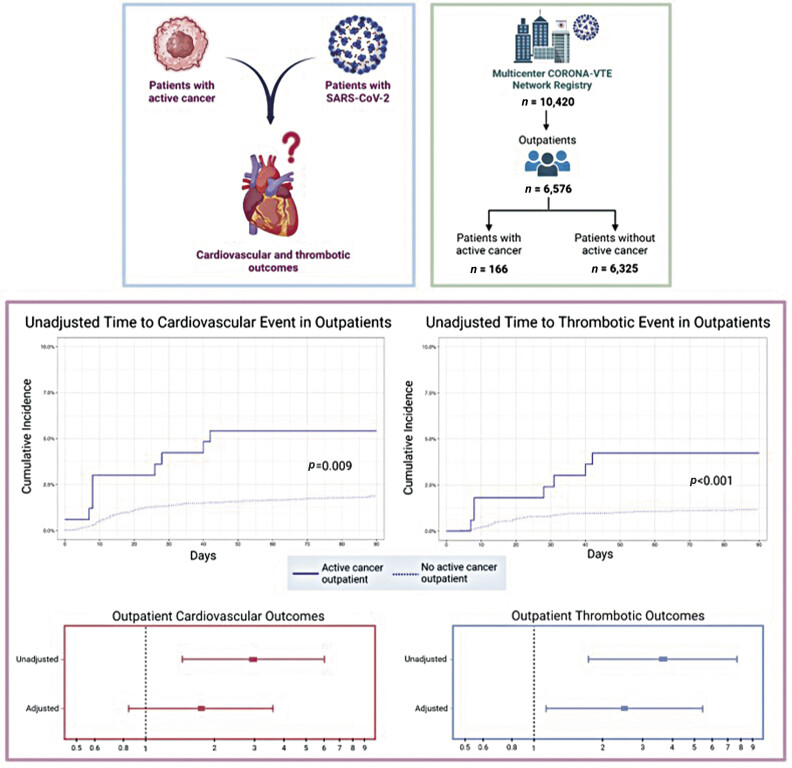
Risk of thrombotic and cardiovascular outcomes among outpatients with COVID-19 and active cancer. COVID-19, coronavirus disease 2019.

In this large, multicenter registry study, outpatients with COVID-19 and active cancer experienced increased incidences of thrombotic and cardiovascular events compared with outpatients with COVID-19 without active cancer ( Fig. 4 ). At 90-day follow-up, the cumulative incidences of thrombotic and cardiovascular events in outpatients with COVID-19 and active cancer were 4.2% and 5.4%, respectively. Corresponding incidences for outpatients with COVID-19 without active cancer were 1.2% and 1.9%. After adjusting for covariates, the risk of thrombotic events was nearly 2.5-fold higher in outpatients with active cancer and COVID-19 compared with those without cancer. However, the difference in hazard of cardiovascular outcomes was not statistically significant after adjustment.

To our knowledge, this is the first paper to show an increased risk of thrombotic events in the outpatient population with active cancer and concomitant COVID-19. One prior study investigated the risk of thrombotic and cardiovascular outcomes in hospitalized and non-hospitalized patients with both cancer and COVID-19 and found that outpatient status was not correlated with an increased risk of VTE. 20 Previous research studies investigating thrombotic outcomes in hospitalized patients with COVID-19 and active cancer presented conflicting results but primarily reported no significant difference in the cumulative incidence of VTE in patients with versus without active cancer ( Table 3 ). 12 13 21 22 23 24 25 26 27 There is likely an increased risk of thrombotic events but not cardiovascular events in the adjusted analysis because inflammation is directly associated with thrombosis and present in cancer and COVID-19. 28 Alternatively, not all cardiovascular events may be directly linked to inflammation, such as arrhythmia and heart failure. 29 However, those results may be affected by the retrospective and single-center nature of the trials, small sample sizes, broad definition of active cancer (diagnosis within the past 5 years), inclusion of patients with a history of cancer, and short-term follow-up.

**Table 3:** Summary of prior studies investigating cardiovascular and thrombotic outcomes in patients with COVID-19 with versus without active cancer

Study	Design	Patient population	*N*	Findings
Alpert et al, 2020 21	Retrospective cohort study	Inpatients with COVID-19 with and without active cancer	5,556 patients; 421 with active cancer	Patients with COVID-19 and concomitant active cancer were more likely to develop VTE compared with those without active cancer (adjusted OR: 1.77, 95% CI: 1.01–3.09)
Patell et al, 2020 13	Retrospective cohort study	Inpatients with COVID-19 with and without active cancer	398 patients; 45 with active cancer	There was no difference in the cumulative incidences of thrombotic events between patients with active cancer (18.2%, 95% CI: 10.2–27.9%) and those without cancer (14.2%, 95% CI: 4.7–28.7%)
Obispo et al, 2020 25	Retrospective cohort study	Inpatients with COVID-19 with and without active cancer	1,127 patients; 86 with active cancer	There was no significant difference in the cumulative incidences of thrombotic events between patients with active cancer (9.8%) and those without cancer (5.8%; *p* = 0.25).
Onder et al, 2023 39	Retrospective cohort study	Inpatients with COVID-19 with and without active cancer	538 patients; 61 with active cancer	Active cancer was an independent predictor of the development of acute ischemic events.

Abbreviations: CI, confidence interval; OR, odds ratio; VTE, venous thromboembolism.

The present analysis may serve to inform future studies aimed at identifying the efficacy of thromboprophylaxis for the prevention of cardiovascular events in outpatients with active cancer and concomitant COVID-19, as well as the association between such events and cancer-directed treatment. Many patients with COVID-19 are treated in outpatient settings, an observation that is likely attributed to extensive vaccine availability and the increasing number of COVID-specific therapies. 30 Due to the lack of evidence, current guidelines do not endorse the routine use of prophylactic anticoagulation for COVID-19 in outpatient settings. 31 32 Trials designed to investigate the efficacy of various prophylactic regimens in outpatients (regardless of active cancer status) were stopped prematurely primarily due to enrollment challenges and have suggested that VTE prophylaxis did not reduce the rates of thrombotic or cardiovascular outcomes, hospitalization, or death. 14 30 33 34 35 In light of our study, further research into the potential role of thromboprophylaxis in outpatients with COVID-19 and active cancer may be warranted. Similarly, previous studies have documented that the incidence of VTE was higher among COVID-19 patients who had received recent systemic cancer-directed therapy. 24 26 36 37 Although our analysis was not dedicated to investigating the association between cancer treatments and adverse events, further research should be conducted to understand the risk associated with various cancer-directed treatments on cardiovascular and thrombotic outcomes in patients with COVID-19.

Our study must be interpreted within the context of its design and limitations. Patient inclusion for this study spanned from 2020 through 2022. Given the evolving nature of COVID-19 and its treatment, the extent to which the results can be extrapolated to newer viral strains requires study. Further, the retrospective nature of this study may have led to missing data and prevented accurate recording of variables. Although the registry included 10,420 patients, it is limited to the five states in which the six centers were located (Massachusetts, Pennsylvania, Virginia, Colorado, and Maryland). Patients in other states may have had dissimilar outcomes due to variations in baseline characteristics, as well as accessibility and quality of health care. However, it is unlikely that such potential differences will be an effect modifier for the observed association. The current study sample includes a large number of patients across age, sex, and ethnoracial groups. However, inpatients were excluded from the final analysis because they are exposed to varying degrees of VTE risk driven by factors related to hospitalization (e.g., need for critical care), severity of COVID-19, comorbid conditions, and immobility 38 such that their thrombotic risk may not be substantively further modified by cancer status. Due to the exclusive focus on outpatients, reliable medication histories were difficult to obtain for all participants. As a result, we were unable to determine the prevalence of thromboprophylaxis in the study population and cannot assess the potential confounding effect on the association between active cancer and the outcomes. The broad definition of active cancer may have captured data from patients with varying degrees of thrombotic risk, limiting our ability to determine whether some particular forms of cancer pose a greater risk for thrombotic and cardiovascular events. Access to the data regarding cancer type and treatment was only available for patients treated at MGB, and only these broad categories were captured, as it was not feasible to capture more granular information. Further, we were unable to quantify the relative contributions of cancer and COVID-19 to the thrombotic and cardiovascular risk in the study cohort. However, our focus was primarily to assess the combination of cancer and COVID-19 on risk relative to patients with only COVID-19. Although fewer than 6% of patients were lost to follow-up, the follow-up period was limited to 90 days post-COVID diagnosis, which may have resulted in an underestimation of thrombotic and cardiovascular events. Lastly, since the identification of thromboembolic events depended on physician assessment of the patients' symptoms and ensuing imaging reports (opposed to asymptomatic screening of all subjects), the true incidences of thrombotic outcomes may be higher than those documented in our results.

Our analysis has some strengths as well. The stringent requirement of a positive RT-PCR test ensured that all participants had objectively confirmed COVID-19. Independent adjudication of outcomes ensured the validity of all reported outcomes, and all patient data were ascertained from electronic health records by trained researchers. Lastly, the multicenter nature of the registry helped to capture a more diverse study population from five states across the United States.

## Conclusion

Our study suggests that outpatients with active cancer and COVID-19 may be at an increased risk of experiencing thrombotic events. However, the evolution of COVID-19 strains and vaccinations has changed the epidemiology of the disease and should be addressed in future research by analyzing patient outcomes within timeframes organized by vaccine prevalence and according to COVID-19 strain. Further research is needed to validate our results and evaluate whether the use of prophylactic anticoagulation in this specific subpopulation is beneficial. Our findings contrast with those of previous studies and may provide a foundation for further investigation and ultimately inform efforts to mitigate the risk of thrombotic and cardiovascular outcomes in this vulnerable outpatient population.

## Notes

**What is Known About This Topic?**

- Active cancer and COVID-19 are each independently associated with an increased risk of cardiovascular and thrombotic events; however, the association between concomitant active cancer and COVID-19 with such events is not well-established.
- Findings from prior studies conducted on hospitalized patients with COVID-19 are conflicting, but primarily observed no significant difference in the incidence of cardiovascular and thrombotic events in those with versus without active cancer.

**What Does This Paper Add?**

- Outpatients with COVID-19 and active cancer experienced increased frequencies of thrombotic and cardiovascular events compared with outpatients with COVID-19 without active cancer.
- After adjusting for potential covariates, the risk of thrombotic events was nearly 2.5-fold higher in outpatients with active cancer and COVID-19 compared with those without cancer.
