# Supplementary material for: Association between Active Cancer and Risk of Thrombotic and Cardiovascular Outcomes in Outpatients with COVID-19: A CORONA-VTE Network Analysis
Source: TH Open. 2025 Oct 17;9:a27132715. doi: 10.1055/a-2713-2715 (PMC12551654; doi:10.1055/a-2713-2715)
Supplement: Supplementary file 1 — Supplementary Material [file 10-1055-a-2713-2715_27262886.pdf]

**Table S1**. Individual components of composite VTE, arterial thrombotic, and cardiovascular events in outpatients with and without active cancer at 90-day follow-up.		
**Outcome**	**Outpatients without active cancer (n = 6,325)**	**Outpatients with active cancer (n = 166)**
Thrombotic outcome composite a (%) [95% CI]	1.2 [0.9-1.5]	4.2 [2.1-8.7]
DVT (%) [95% CI]	26.0 [17.3-37.1]	28.6 [9.5-63.2]
PE (%) [95% CI]	30.1 [21.0-41.3]	28.6 [9.5-63.2]
Superficial vein thrombosis (%) [95% CI]	9.6 [5.1-18.1]	14.3 [3.7-50.5]
Myocardial infarction b (%) [95% CI]	2.7 [0.0-10.1]	0 [0.0-17.8]
Stroke (%) [95% CI]	11.0 [6.0-20.0]	0 [0.0-17.8]
Transient ischemic attack (%) [95% CI]	1.4 [0.3-7.4]	14.3 [3.7-50.5]
Catheter related thrombosis (arterial) (%) [95% CI]	1.4 [0.3-7.4]	0 [0.0-17.8]
Major adverse limb event (%) [95% CI]	5.5 [2.7-12.8]	0 [0.0-17.8]
Cardiovascular composite c (%) [95% CI]	1.9 [1.6-2.2]	5.4 [2.9-10.3]
Newly diagnosed heart failure or heart failure hospitalization (%) [95% CI]	10.3 [6.7-15.7]	0 [0.0-14.9]
New atrial fibrillation (%) [95% CI]	25.9 [19.8-32.9]	22.2 [11.5-49.4]
Myocarditis (%) [95% CI]	0.9 [0.6-3.4]	0 [0.0-14.9]
Cardiovascular related death (%) [95% CI]	3.4 [1.8-7.1]	11.1 [8.3-37.0]
All-cause mortality (%) [95% CI]	1.6 [1.3-1.9]	7.8 [4.6-13.0]

The frequencies of the individual components of composite thrombotic and composite cardiovascular events represent the percentage of each respective event within the total number of each composite, rather than the frequencies of these events across the entire cohort. All outcomes were adjudicated by independent physicians using uniform pre-defined criteria.

[a] Includes composite VTE and arterial thrombotic events.
[b] Only Type I myocardial infarction was collected.
[c] Includes composite thrombotic events.

Abbreviations: CI = confidence interval; DVT = deep vein thrombosis; PE = pulmonary embolism; VTE = venous thromboembolism.

**Table S2.** Cumulative incidence rates of composite thrombotic and cardiovascular events at day 90 among outpatients with and without active cancer after exclusion of superficial vein thrombosis.	
**Outcomes**	**Sensitivity Analysis Cumulative Incidence Rate % (95% Cl)**
Active Cancer	
Thrombotic Outcome Composite	3.6 (1.65-7.95)
Cardiovascular Outcome Composite	5.4 (2.88-10.26)
No Active Cancer	
Thrombotic Outcome Composite	1.1 (0.85-1.37)
Cardiovascular Outcome Composite	1.9 (1.56 – 2.24)

Abbreviation: CI = confidence interval.

TH Open | DOI 10.1055/a-2713-2715 | © The Author(s).
